# Analysis of setup error based on CTVision for nasopharyngeal carcinoma during IGRT

**DOI:** 10.1120/jacmp.v17i4.6083

**Published:** 2016-07-08

**Authors:** Shuxu Zhang, Xiang Zhou, Quanbin Zhang, Shaohui Jiang, Ruihao Wang, Guoqian Zhang, Huaiyu Lei, Shengqu Lin

**Affiliations:** ^1^ Radiotherapy Center, Cancer Center of Guangzhou Medical University, Affiliated Tumor Hospital of Guangzhou Medical University Guangzhou China

**Keywords:** nasopharyngeal carcinoma, setup errors, image‐guided radiotherapy (IGRT), reconstructive thickness

## Abstract

The aim of the present study was to investigate the role of CTVision in interfractional setup errors during intensity‐modulated radiation therapy (IMRT) in 12 nasopharyngeal carcinoma (NPC) patients. The trend of setup errors as a function of time during a fractionated radiotherapy course was investigated, and the influence of reconstructive thickness on image reconstruction for setup errors was analyzed. The appropriate planning target volume (PTV) margin and planning risk volume (PRV) margin were defined to provide a reference for the design of IMRT for NPC. Based on CTVision, online CT was performed weekly for each patient. Setup errors were measured by registration between the CT reconstructed image and reference image. Mean of setup errors, estimated population systematic (:), and population random (σ) errors were calculated using SPSS (v15.0). Optimum PTV and PRV margins were calculated. In the clinical data, for the LR (left–right), SI (superior–inferior), and AP (anterior–posterior) directions, : was 0.8, 0.8, and 1.0 mm, respectively, and σ was 1.0, 1.3, and 0.8 mm, respectively. In the LR, SI, and AP directions, PTV margins were at least 2.7, 2.9, and 3.0 mm, respectively, and PRV margins were at least 1.5, 1.7, and 1.7 mm, respectively. No significant differences in setup errors were observed during the fractionated radiotherapy course (p>0.05). However, CT image reconstruction with different thicknesses affected the accuracy of measurements for setup errors, particularly in the SI direction. The application of CTVision to correct setup errors is important and can provide reasonable margins to guarantee the coverage of PTVs and spare organs at risk. A thickness of 3 mm in the reconstructed image is appropriate for the measurement of setup errors by image registration.

PACS number(s): 87.55.Qr

## I. INTRODUCTION

Nasopharyngeal carcinoma (NPC) is a common malignancy in Asia, especially in Southern China. Currently, intensity‐modulated radiation therapy (IMRT) or comprehensive treatment based on IMRT is the main treatment method for NPC, and it can improve tumor control and decrease radiation induced complications.^(1)^ However, the reproducibility of patient positioning with accurate immobilization devices is the key to ensure the efficacy of IMRT.^(2)^ Although there are several immobilization devices for patient positioning, such as a thermoplastic mask, vacuum cushion, and individual headrest, setup errors are inevitable during fractionated radiotherapy.^(3)^

Monitoring and correcting setup errors is an important part of quality control in radiotherapy. At present, the most effective method to reduce setup error is image‐guided radiotherapy (IGRT), mainly with a megavoltage (MV) electronic portal imaging device (EPID),^(4)^ MV cone‐beam CT (MV‐CBCT),^(5)^ kilovolt CBCT (KV‐CBCT),^(5)^ and KV orthogonal X‐ray images.^(6)^ However, the sharpness, contrast, and resolution of images obtained with the above IGRT methods are not as good those of conventional CT images, resulting in difficulties in the timely modification of radiotherapy planning programs according to changes in gross target volume (GTV) and surrounding normal tissues, namely adaptive radiotherapy (ART). From the perspective of IGRT or ART, the integration models between CT and linac, such as the CT‐on‐rail accelerator system (CTVision) (Siemens AG, Berlin, Germany), have a unique advantage. The CTVision is equipped with an online CT scanner (SOMATOM Sensation Open CT, Siemens AG, Berlin, Germany) with a 40‐row detector and 82 cm large aperture. More importantly, online CT and linac have a shared treatment couch. Before radiotherapy, it is available for 4D CT scanning or conventional 3D CT acquisition; furthermore, the CT images can be acquired in the radiotherapy position. The registration can then be performed with the reference CT images to monitor and correct setup errors and improve the accuracy of radiotherapy. In addition, it facilitates treatment planning and modifications according to GTV changes, enabling ART based on online CT. In the present study, 12 patients with NPC who were treated with IMRT underwent weekly fan‐beam kV‐CT scanning based on CTVision. The acquired CT images were registered with reference images. According to the differences identified by registration, setup errors were corrected in three directions. The clinical target volume (CTV) to planning target volume (PTV) and organs at risk (OAR) to planning risk volume (PRV) margins were calculated to provide a reference for the design of IMRT. In addition, the trends of setup errors as a function of time during the fractionated radiotherapy course were investigated**.** The effect of thickness on CT image reconstruction for setup errors was also examined.

## II. MATERIALS AND METHODS

### A. Patient group

Twelve consecutive patients (eight men and four women) with nonmetastatic NPC treated with IMRT at our institution between May 2015 and July 2015 were included in the study. The average age of patients was 44.4 years (range, 29–62 years). According to the 2002 Tumor Node Metastasis (TNM) classification, patient stages were as follows: Stage I in one patient (8%), Stage II in three (25%), Stage III in seven (59%), Stage IV in one (8%). Before radiotherapy, all patients signed informed consent forms.

### B. Acquisition of planning CT images

Patients were immobilized in the supine position using a customized thermoplastic mask secured to a carbon fiber plate with four clamp bases covering the head and neck. The carbon fiber plate was placed on the linac treatment couch shared with online CT of CTVision. Positioning scans were performed by online CT of CTVision. The slice thickness was 3 mm and the matrix was 512×512. The CT images were transferred to the Pinnacle System (V9.0, Philips Medical Systems, Fitchburg, WI), and target volumes were drawn on each CT image according to ICRU recommendations. Finally, the treatment planning of IMRT was designed by physicists.

### C. Positioning reference marking

After the completion of radiation treatment planning and dose verification, treatment planning was confirmed using a Simulix‐Evolution X‐ray simulator (Nucletron B.V., Veenendaal, The Netherlands). The isocenter was confirmed by moving the couch of the X‐ray simulator based on the parameters of treatment planning. After that, the “+” crosslines were labeled on the thermoplastic mask in the right, left, and anterior directions, which coincided with the “+” crosslines from laser. The “+” crosslines were taken as the reference mark for positioning. But some potential sources of errors, such as laser alignment between linac and X‐ray simulator, display accuracy and isocenter accuracy, were not taken into consideration.

### D. Measurement of setup errors in fractionated radiotherapy

Before the first radiotherapy session, the patients were positioned on the treatment couch of CTVision according to the reference marks. Then, the treatment couch was rotated 180° for online CT scanning, and the parameters of the scan were consistent with those of planning CT. In this way, CT images were collected under the radiotherapy position, which served as floating images for the comparison with the reference images (initial planning CT images). After the first radiotherapy session, online imaging for correction of setup errors by CTVision was performed weekly for each patient by two technicians with more than five years of experience. After the registration between floating images and reference images, setup errors were recorded as translational deviations of the isocenter along the three axes, namely left–right (LR), superior–inferior (SI), and anterior–posterior (AP). Setup errors in the right, superior, and anterior directions were defined as positive values, whereas the others were negative. If the setup error was >3 mm, setup errors were corrected by adjusting the treatment couch to match the treatment isocenter. Images were analyzed by two radiation oncologists with adequate training to reduce interobserver variability. Only interfractional setup errors were considered in this study, while intrafractional setup errors and organ motion were not assessed.

### E. Systematic and random setup errors

For each patient, the systematic error in each direction was calculated as the mean of all setup errors recorded during the fractionated radiotherapy course, while the random error in each direction was the standard deviation (SD) of the patient setup errors. The populations' mean of setup errors was the average of all patients' means in each direction. The population systematic error (:) was expressed as the SD of the patient systematic errors, while the population random error (σ) was the root mean square of the average of all individual SDs.^(7)^

### F. Evaluation of PTV and PRV margins

The CTV to PTV margin was calculated according to the equation: $M_{\text{PTV}}=2.5\Sigma +0.7\sigma$, with more than 99% of CTV receiving at least 95% of the prescribed dose,^(8)^ and the OAR to PRV margin was calculated by the equation: $M_{\text{PRV}}=1.3\Sigma +0.5\sigma$.^(9)^ The impact of both systematic and random setup errors was taken into account in the equation. The estimation was performed in all directions.

### G. Influence of reconstructive thickness on setup errors

The CT images obtained in the radiotherapy position were reconstructed with different thickness (2, 3, and 5 mm) and registered with the reference images, which were also reconstructed in the corresponding thicknesses. Setup error was recorded again respectively. The aim was to investigate the influence of reconstructive thickness on setup errors.

### H. Statistical analysis

The statistical analysis of setup errors was performed by SPSS 19.0 statistical software (IBM SPSS, Armonk, NY). A *t*‐test was used to compare differences in each direction, and one‐way analysis of variance was used to analyze the changes in setup errors as a function of time and reconstructive thickness. The difference was considered significant when p<0.05.

## III. RESULTS

### A. Analysis of setup error

**Figure 1:**
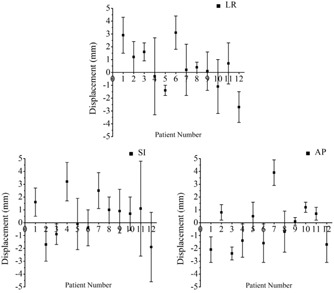
The distribution of all setup errors in three directions obtained from CT reconstructed images with 3 mm thickness.

**Figure 2:**
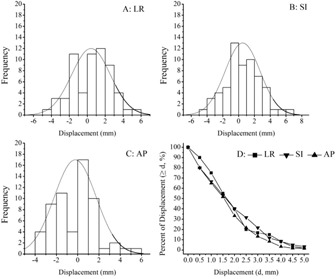
Normal distributions of all setup errors: (a) left–right, (b) superior–inferior, and (c) anterior–posterior directions. The absolute frequency of setup error greater than a set value is shown in (D).

Setup error refers to the deviation of displacement between actual radiotherapy position and radiotherapy planning position. A small error in positioning results in a better reproducibility of the position. The distribution of all setup errors in three directions is shown in Fig. 1. Regardless of the duration of the fractionated radiotherapy course, the setup errors in the LR, SI, and AP directions were 0.41±2.16,0.50±2.22, and −0.24±1.97 mm, respectively, without significant differences among them (p>0.05). These results are consistent with the requirement that the ideal setup error should be <2.0 mm for head and neck cancer. Setup errors are normally distributed in the LR, SI, and AP directions, as shown in Fig. 2, while the setup error in 95% confidence interval was 3.7, 3.3, and 3.7 mm in the corresponding directions. The absolute frequency distributions, which were greater than the fixed value for the three directions, are also displayed in Fig. 2. The setup errors exceeding 5.0 mm were approximately 1.67%, 3.33%, and 1.67% in the LR, SI, and AP directions, respectively, and approximately 83.33%, 78.33%, and 86.67%, respectively, for the setup errors of ≤3.0 mm.

### B. Systematic and random setup error

Setup error includes systematic and random error.^(10^) The systematic error, such as the mechanical error of CT and linac, and the error of laser, represents displacement that is present during the entire course of treatment. The random error, caused by the operation of technicians or motion of organs, represents daily variation in the setup of a patient. In this study, the population systematic error (:) was 0.8, 0.8, and 1.0 mm in the LR, SI, and AP directions, respectively, while the population random error (σ) in the corresponding directions was 1.0, 1.3, and 0.8 mm, respectively.

### C. Time trends

**Figure 3:**
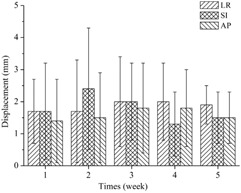
The setup errors in three directions during the fractionated radiotherapy course.

The setup errors in the three directions as a function of time during the fractionated radiotherapy course are shown in Fig. 3. There were no statistically significant changes in time‐trends during the fractionated radiation therapy course (p>0.05), which is consistent with the results reported by Mongioj et al.^(4^) In terms of accuracy and repeatability, immobilization with a customized thermoplastic mask was reliable, without obvious changes in patient position over time.

### D. PTV and PRV margin calculation

Based on the equations $M_{\text{PTV}}=2.5\Sigma +0.7\sigma$ and $M_{\text{PRV}}=1.3\Sigma +0.5\sigma$, the PTV margins should be at least 2.7, 2.9, and 3.0 mm for the LR, SI, and AP directions, respectively, while the PRV margins in the corresponding directions should be at least 1.5, 1.7, and 1.7 mm, respectively. The PTV margins obtained in this study were within the range of the margins for treatment planning.

### E. Influence of reconstructive thickness on setup error

Regardless of the duration of the fractionated radiotherapy course, the setup error obtained from the registration between reference image and CT image reconstruction with a reconstructive thickness of 2, 3, and 5 mm, respectively, is shown in Table 1, and these values were normally distributed in the LR, SI, and AP directions, respectively. In terms of the influence of reconstructive thickness on setup error, there were no significant differences in the setup errors in the LR and AP directions (p>0.05). However, in the SI direction, the setup error obtained from the reconstructed image with 5 mm thickness showed a wider deviation, which was not observed for the 2 and 3 mm thicknesses; moreover, it was significantly different from that obtained from the reconstructed image with 2 mm or 3 mm thickness (p<0.05). However, the setup errors did not differ significantly between the reconstructive thicknesses of 2 and 3 mm for image reconstruction (p>0.05). As shown in Table 2, with the change in thickness, the percentages of setup errors for >5.0 mm and ≤3.0 mm in the LR and AP directions were not significantly different, whereas in the SI direction, the percentages of setup errors for ≤3.0 mm obtained from both 2 and 3 mm of reconstructive thickness was approximately 83.33%, which is larger than that obtained from 5 mm of reconstructive thickness by approximately 33.33%. In addition, when the reconstructive thickness was 5 mm, setup errors exceeding 5.0 mm were found more frequently than those observed for a thickness of 3 mm (approximately 23.33%). On the other hand, because the results of $\Sigma ,\sigma ,M_{\text{PTV}}$, and $M_{\text{PRV}}$ were directly based on the setup error, the effects of reconstructive thickness were similar to those of setup error, as shown in Table 3.

**Table 1:** The setup errors in three directions with the change in reconstructive thickness

	*Deviation in Different Reconstructive Thicknesses (mm)*
*Direction*	*2 mm*	*3 mm*	*5 mm*
LR	0.26±1.94 ^a^	0.41±2.16 ^a^	0.44±2.09 ^a^
SI	0.52±1.83 ^a^	0.50±2.22 ^a^	1.31±4.18 ^b^
AP	−0.11±2.03 ^a^	−0.24±1.97 ^a^	−0.20±2.17 ^a^

a ^a^ After the pairwise comparisons between different thicknesses in the same direction, there no significant difference for p>0.05.

b ^b^ The difference is statistically significant for p<0.05.

**Table 2:** The percentages of setup errors greater than or less than or equal to a set value in three directions with the change in reconstructive thickness

*Reconstructive Thickness*	*LR*		*SI*		*AP*	
	≤3.0 mm	>5.0 mm	≤3.0 mm	>5.0 mm	≤3.0 mm	>5.0 mm
2 mm	86.67%	0.00%	88.33%	0.00%	83.33%	0.00%
3 mm	83.33%	1.67%	78.33%	3.33%	86.67%	1.67%
5 mm	85.00%	3.33%	33.33%	23.33%	78.33%	1.67%

**Table 3:** The $\Sigma ,\sigma ,M_{\text{PTV}}$, and $M_{\text{PRV}}$ values in three directions with the change in reconstructive thickness

*Reconstructive Thickness*	*LR*				*SI*				*AP*			
	*:*	*σ*	$M_{PTV}$	$M_{PRV}$	*:*	*σ*	$M_{PTV}$	$M_{PRV}$	*:*	*σ*	$M_{PTV}$	$M_{PRV}$
2 mm	0.6	1.0	2.3	1.3	0.6	0.9	2.3	1.3	1.0	0.9	3.0	1.7
3 mm	0.8	1.0	2.7	1.5	0.8	1.3	2.9	1.7	1.0	0.8	3.0	1.7
5 mm	0.8	1.1	2.6	1.5	1.3	1.9	4.6	2.7	1.0	0.9	3.2	1.8

All units are mm.

## IV. DISCUSSION

### A. Analysis of setup error

Radiation therapy is the main treatment method for NPC; however, setup error during the course of radiotherapy is inevitable and has a significant impact on the efficacy of radiation therapy.^(3^) How to reduce setup errors has attracted the attention of researchers. A study on the treatment of head and neck cancer with CBCT showed that the rate of setup error exceeding 5.0 mm was more than 11%, and 29% of the setup error was >3.0 mm without IGRT; furthermore, in 15% to 31% of patients, setup errors need to be corrected by IGRT during the course of radiotherapy.^(11^) However, setup errors are significantly reduced with the increase in frequency of IGRT. The results of the present study indicate ≤3.33% of setup error exceeding 5.0 mm and 21.77% of setup error >3.0 mm, suggesting that the deviation of setup error obtained by CTVision is smaller than that obtained by CBCT. A study focusing on setup error in head and neck cancer using EPID concluded that : and σ were approximately 1.6–4.6 mm and 1.1–2.5 mm, respectively,^(12,13^) which was higher than that the values obtained in the present study, (0.8–1.0 mm and 1.0–1.3 mm). The : and σ values obtained from setup error in NPC using kV‐CBCT were 1.1–1.3 mm in all directions in all patients.^(14^) The : and σ observed in the present study were slightly smaller than these values.

Rapid and high‐contrast diagnostic CT imaging for treatment planning was introduced into the radiotherapy room by CTVision, which can provide an advantage to effectively measure and correct setup error during a fractionated radiotherapy course.^(15^) Furthermore, the thermoplastic masks and immobilization devices that are used in our department were previously used in IMRT delivery for head and neck cancer to improve the reproducibility and stability of patient position, successfully maintaining setup error under 2.0 mm in all patients.^(16^) It should be worth noting that minor undetectable setup errors might be caused by couch rotation between CT‐on‐rail and the linac.^(17^) According to the previous studies, the geometric accuracy of the treatment couch in integrated CT‐linac combinations is ≤1 mm after a rotation, or even less than 0.5 mm.^(18,19^) If compared with CBCT or EPID, the accuracy to monitor setup errors by CTVsion is needed additional clinical data to verify when considering more uncertainty.

### B. Time trends

As reported previously, patient weight loss or tumor shrinkage reduce neck thickness during the treatment course. This results in significant anatomical changes and increases the setup error over time, which negatively affects the efficacy of IMRT.^(20^) However, there was no significant difference in setup errors during the fractionated radiotherapy course in our study, consistent with previously reported data.^(21^) In this study, a customized thermoplastic mask covering the head and neck of the patient was fixed to the treatment couch of the linac with four fixation points and without any base plate attachment. Furthermore, the customized mask is fitted to the facial skin of patients. On the other hand, bony landmarks of the head and neck do not obviously change during weight loss or tumor shrinkage, remaining consistent with the spatial location of the mask without significant loosening of the immobilization device. In our study with small populations, there were no obvious changes in facial contours in approximately 60% of patients during radiation treatment. The experienced technicians ensured that the accuracy and reproducibility of immobilization would be effectively maintained during the entire course of fractionated radiotherapy. However, whether the mask needs to be recustomized during the course of fractionated radiotherapy, especially in the late stages, needs to be determined with additional clinical data in future studies.

### C. Evaluation of PTV and PRV margins

Currently, the PTV margin is based on a certain range of expansion for CTV and the foreseeable setup errors and organ motion. A reasonable PTV margin plays an important role in the advantage of IMRT. If the PTV margin is narrow, the target volume may be missed. By contrast, if the PTV margin is wide, it can lead to excessive exposure of normal tissues, which cannot be effectively protected. Regarding the treatment of NPC, a reduction in the PTV margin of 1.0 mm is correlated with an average reduction in the dose to the parotid of 1.3 Gy.^(22)^ A narrow PTV margin would provide effective protection of the parotid. Therefore, accurate measurement and correction of setup error is the key to ensure and control the quality of radiotherapy. To ensure a sufficiently narrow PTV margin to cover the target volume completely, if the correction of setup error is carried out daily by CTVision, the patients will receive additional radiation doses and the workload for technicians will be increased. Therefore, it is not widely suitable for clinical practice. In this study, the PTV and PRV margins accounting for setup errors were approximately 2.7–3.0 mm and 1.5–1.7 mm, respectively. Monitoring and correcting setup error in small populations is performed by CTVision to obtain a reasonable and clinically significant PTV margin and PRV margin, which can provide a theoretical basis for a reasonable design of IMRT for NPC in our department. However, interfractional setup error is not the only consideration for margins and there are many components that need to be considered such as intrafraction setup error, organ motion, image registration, uncertainties of treatment planning, and transfer errors from the planning CT to the simulator. At the same time, the PTV and PRV margins obtained in this study only provide a reference because of the limited number samples. Further analysis with a larger number of samples will provide additional data with clinical significance.

### D. Influence of reconstructive thickness on setup error

Reconstructive thickness is acquired during data processing and does not affect the scanning time; it only changes the reconstructed time and frames of the reconstructed image. Reducing reconstructive thickness not only reduces the effects of partial volume, but also improves image quality of MPR (multiplanar reformation) and three‐dimensional reconstructed images, such as MIP (maximum intensity projection), SSD (shaded surface display), and VR (volume rendering).^23^, ^24^ Theoretically, the reduction of thickness would improve the quality of CT image reconstruction, which could reduce the deviation in the measurement of setup error by CT image registration, resulting in a more realistic setup error. However, an increase in the time of reconstruction and reconstructed image frames will make the practical work inconvenient. Thus, it needs to be optimized.

When the slice thickness is 3 mm in the CT scan, a reconstructive thickness of 2 mm can reduce the effects of partial volume compared with 3 mm in reconstructive thickness. However, there was no statistically significant difference between the setup errors obtained with 2 mm and 3 mm of reconstructive thickness. With 2 mm of reconstructive thickness, the data processing time will be increased, and the space for image storage will be increased as well as the workload of delineating target volumes. The results of the present study indicate that increasing the thickness for CT image reconstruction has a significant effect on setup error, especially in the SI direction. The changes in reconstructive thickness affect the partial volume effect in the longitudinal axis, namely the SI direction, which affects the quality of the postprocessing image. Spatial resolution in the longitudinal axis is an important factor determining the quality of the reconstructed image, and reducing the thickness is important for space resolution in the longitudinal axis.^25^, ^26^ The increase of reconstructive thickness would reduce the spatial resolution of CT image reconstruction, leading to a wider deviation of setup error by image registration. Therefore, it is more appropriate to use 3 mm of reconstructive thickness for image reconstruction to measure the setup error by image registration.

## V. CONCLUSION

The present results highlight the importance of CTVision to monitor and correct setup errors and show that it provides a more realistic setup error than that obtained by EPID or CBCT. Reasonable margins can improve accuracy, resulting in better coverage of PTVs and sparing organs at risk, thus minimizing the irradiation of normal tissues. Because of the small population of the study, the trend of setup error as a function of time was not significant, and this needs to be examined with additional clinical data in future studies. It is more appropriate to use a reconstructive thickness of 3 mm for image reconstruction to measure setup error by image registration.

## ACKNOWLEDGMENTS

This work has been financially supported by Science and Technology Project of Guangdong Province (grant no. 2013B021800274), and Science and Technology Innovation Project of Education Department in Guangdong Province (grant no. 2013KJCX152), as well as Featuring Innovation Project of Education Department in Guangdong Province (grant no. 2014KTSCX104).

## COPYRIGHT

This work is licensed under a Creative Commons Attribution 3.0 Unported License.
